# Regulatory Mechanisms of Mitogen-Activated Protein Kinase Cascades in Plants: More than Sequential Phosphorylation

**DOI:** 10.3390/ijms23073572

**Published:** 2022-03-25

**Authors:** Haigang Ma, Yujiao Gao, Yonggang Wang, Yi Dai, Hongxiang Ma

**Affiliations:** Jiangsu Key Laboratory of Crop Genomics and Molecular Breeding/Jiangsu Co-Innovation Center of Modern Production Technology of Grain Crops/Joint International Research Laboratory of Agriculture and Agri-Product Safety, The Ministry of Education of China, Yangzhou University, Yangzhou 225009, China; yujiaogao@yzu.edu.cn (Y.G.); yg.wang@yzu.edu.cn (Y.W.); daiyi@yzu.edu.cn (Y.D.)

**Keywords:** MAPK, phosphorylation, activation, development, innate immunity, abiotic stress

## Abstract

Mitogen-activated protein kinase (MAPK) cascades play crucial roles in almost all biological processes in plants. They transduce extracellular cues into cells, typically through linear and sequential phosphorylation and activation of members of the signaling cascades. However, accumulating data suggest various regulatory mechanisms of plant MAPK cascades in addition to the traditional phosphorylation pathway, in concert with their large numbers and coordinated roles in plant responses to complex ectocytic signals. Here, we highlight recent studies that describe the uncanonical mechanism of regulation of MAPK cascades, regarding the activation of each tier of the signaling cascades. More particularly, we discuss the unusual role for MAPK kinase kinases (MAPKKKs) in the regulation of MAPK cascades, as accumulating data suggest the non-MAPKKK function of many MAPKKKs. In addition, future work on the biochemical activation of MAPK members that needs attention will be discussed.

## 1. Introduction

The mitogen-activated protein kinase (MAPK) cascade is a three-tiered system composed of three protein kinases: MAPK kinase kinase (MAPKKK), MAPK kinase (MAPKK), and MAPK [1]. These are serine/threonine protein kinases, which phosphorylate serine (S) or threonine (T) residues in conserved motifs of target proteins to modify their functions. The MAPK cascade is conserved in eukaryotes including yeast, mammals, and plants, where it mediates extracellular signal transduction into cellular responses through the three members phosphorylating each other in a serial way, such that MAPKKK phosphorylates and activates MAPKK, which in turn phosphorylates and activates MAPK [1].

Plant genomes encode relatively more MAPK members than yeast and mammal genomes. Over 60 MAPKKKs, 20 MAPKKs, and 10 MAPKs are found in the dicotyledonous model plant Arabidopsis (*Arabidopsis thaliana*) genome [2,3], and 75 MAPKKKs, 8 MAPKKs, and 17 MAPKs in the monocotyledonous rice (*Oryza sativa*) genome [4,5,6]. Therefore, theoretically, a particularly large number of MAPK cascades exist in plants.

MAPK cascades have been reported to be involved in various biological processes in plants, as well as reviewed previously and very recently [7,8,9,10]. Substantial efforts over recent decades have enabled the functional identification of lots of MAPK members and many complete MAPK cascades in regulation of plant development and responses to environmental stimuli. Previous studies mostly focused on sequential phosphorylation when they encountered the MAPK cascades. As a considerable amount of data in plants, including tomato (*Solanum lycopersicum*), Arabidopsis, and rice, indicate uncanonical regulatory mechanisms of MAPK cascades, the elucidation of the underlying mechanism of the activation of MAPK cascades has become increasingly important. In this review, we discuss various alternative regulatory mechanisms of plant MAPK cascades in addition to the traditional phosphorylation pathway.

## 2. Activation of MAPKKKs in MAPK Cascades

The activation of a classical MAPK cascade is initiated by the phosphorylation and activation of MAPKKK. To date, various proteins have been found to activate MAPK cascades, and some of them directly bind MAPKKKs; however, only a few receptor-like kinases (RLKs) have been determined to directly phosphorylate and activate MAPKKKs.

Approximately two decades ago, a complete MAPK cascade that regulates cytokinesis in tobacco (*Nicotiana tabacum*) cells was identified. This signaling cascade, which comprises NPK1 (a MAPKKK), NQK1 (a MAPKK, also known as NtMEK1), and NRK1 (a MAPK, also known as NTF6), regulates cell division through sequential phosphorylation [11,12]. The NPK1-NQK1-NRK1 (PQR) cascade is activated by NACK1 and NACK2, two members of the kinesin-like protein family [13]. NACK1 co-localizes and physically interacts with NPK1; furthermore, its binding increases NPK1 kinase activity [13] (Table 1). However, how NPK1 is activated at the biochemical level remains unclear, although the activation of the PQR cascade is known to be repressed by cyclin-dependent kinases (CDKs), which phosphorylate both NACK1 and NPK1 and, in turn, inhibit their mutual interaction, thus inhibiting the activation of NPK1 [14]. Similarly, the orthologous signaling cascade of the abovementioned PQR pathway in Arabidopsis, ANP1/ANP2/ANP3 (three MAPKKKs)-ANQ (also known as MKK6)-MPK4, also functions downstream of AtNACK1 (also known as HIK) to regulate cytokinesis in a similar fashion [15,16,17,18,19].

RLKs have also been reported to bind to and activate MAPKKK in the MAPK cascade. In Arabidopsis embryogenesis, a receptor-like cytoplasmic kinase (RLCK), SSP, activates the YDA (MAPKKK)-MKK4/MKK5-MPK3/MPK6 cascade to regulate embryonic patterning [20,21] (Table 1). In this case, although it has been established that SSP physically interacts with and activates YDA after zygote formation (Table 1), the biochemical details of YDA activation remain obscure [22].

During cold stress, Arabidopsis CRLK1, a calcium (Ca^2+^)/calmodulin-regulated RLK, activates a potential MAPK cascade mediated by MEKK1 (a MAPKKK) to increase cold responsive genes expression [23,24]. CRLK1 interacts with and phosphorylates MEKK1 [23,24]; however, whether the phosphorylation directly activates MEKK1 is not determined.

In plant innate immunity, MAPK cascades are activated by RLCKs. The first layer of innate immunity, triggered by the perception of pathogen-associated molecular patterns (PAMPs) by plant pattern-recognition receptors (PRRs), is termed pattern-triggered immunity (PTI) [31]. At least two complete MAPK cascades are activated during PTI in Arabidopsis: the MEKK1-MKK1/MKK2-MPK4 and the MAPKKK3/MAPKKK5-MKK4/MKK5-MPK3/MPK6 cascade [25,26,32,33,34,35,36]. Both are activated in response to PAMP treatment and induce immune responses. Furthermore, the activation of these two cascades relies on the direct phosphorylation and activation of the two MAPKKKs by RLCKs. After the recognition of PAMPs by PRRs, the MAPKKKs in the two pathways are directly phosphorylated and activated by RLCKs, leading to activation of the MAPK cascades and the consequent regulation of immune responses [25,26,27] (Table 1). Similarly, two rice MAPKKKs, OsMAPKKK18 (the ortholog of Arabidopsis MAPKKK5) and OsMAPKKK24 (also known as OsMAPKKKε), are also directly phosphorylated and activated by the RLCK OsRLCK185 in response to PAMP treatment (Table 1). The two phosphorylated rice MAPKKKs then activate downstream MAPK cascades consisting of OsMPKK4-OsMPK3/OsMPK6 to induce defense responses [28,29].

The second layer of plant innate immunity is termed effector-triggered immunity (ETI), and is initiated by the direct or indirect interaction between plant intercellular nucleotide-binding leucine-rich repeat receptors (NLRs) and pathogen-secreted effectors [31]. RLCKs are also involved in the activation of MAPK cascades in ETI. In the tomato resistance response to the bacterial pathogen *Pseudomonas syringae* pv. *tomato*, host protein kinases Pto and Fen bind to the pathogen effectors AvrPto or AvrPtoB, and the host NLR Prf binds to Pto and Fen to indirectly recognize pathogen effectors and trigger ETI [37,38,39,40,41]. Consistently, MAPK cascades mediated by MAPKKKα have been reported to contribute to Prf-mediated ETI [42,43,44]. Furthermore, SlMai1, a tomato RLCK, is recently identified as a MAPKKKα-interacting protein that regulates NLR-induced cell death [45]. Although SlMai1 does not show in vitro kinase activity, its physical interaction with MAPKKKα increases downstream MAPK activation [45] (Table 1). Interestingly, SlMai1 kinase activity is not required for its function [45], suggesting an undiscovered mechanism of MAPKKKα activation.

The signaling pathways described here exemplify how a MAPK cascade is activated through the activation of MAPKKK directly or indirectly bound to or phosphorylated by different proteins. However, elucidation of the biochemical basis of MAPKKK activation warrants further research.

## 3. Uncanonical Regulation of MAPK Cascades by MAPKKKs

In the canonical MAPK cascade in plants, MAPKKK typically phosphorylates the downstream MAPKK on the two conserved S and T residues in the S/T-XXXXX-S/T (X represents any amino acid) motif [7]. This activates MAPKK to phosphorylate and activate the downstream MAPK. Therefore, MAPKKK, in a classical MAPK cascade, activates the cascade via its kinase activity toward MAPKK. However, several studies have suggested uncanonical mechanisms underlying the regulation of MAPK cascades by MAPKKKs.

### 3.1. Uncanonical Roles of MEKK-like MAPKKKs in the Regulation of Plant MAPK Cascades

Plant MAPKKKs can be simply grouped into MEKK-like and Raf-like MAPKKKs based on their sequence similarity [1,3]. Most of the MEKK-like MAPKKKs have been reported to exhibit typical MAPKKK features that directly phosphorylate and activate downstream MAPKKs in a MAPK cascade. However, several studies have suggested that MEKK-like MAPKKKs can also function as unconventional MAPKKKs to regulate the MAPK cascade.

In Arabidopsis, a complete MAPK cascade (MEKK1-MKK1/MKK2-MPK4) functions downstream of RLCKs in response to pathogen infection [26,33,34,35]. Disruption of this MAPK cascade results in constitutive defense responses with excessive accumulation of salicylic acid (SA) and hydrogen peroxide (H_2_O_2_) in a MEKK2-dependent manner [30,33,46,47,48,49,50]. MEKK2, a MEKK-like MAPKKK, is a paralog of MEKK1, located in a tandem repeat region consisting of MEKK1, MEKK2, and MEKK3 [46,47]. MEKK2 physically interacts with MPK4 via its amino (N)-terminal domain and directly inhibits the activation of MPK4 triggered by the phosphorylation of MKK2, which is an MAPKK upstream of MPK4 in plant innate immunity [46,51] (Table 2). Interestingly, the carboxy (C)-terminal kinase domain of MEKK2 is responsible for the inhibition of AtMPK4 activation [51]. As the inhibition of MPK4 activation in turn activates plant defense responses, and MEKK2 kinase activity is not necessary for the activation of defense responses, the role of MEKK2 kinase activity in plant biological processes is worth further clarification. As an example, the role of MEKK2 in regulating the MEKK1-MKK1/MKK2-MPK4 cascade illustrates the uncanonical function of MEKK-like MAPKKKs.

Another MEKK-like MAPKKK, MKKK7, is involved in innate immunity in Arabidopsis [52]. Briefly, MKKK7 interacts with FLS2, a RLK that recognizes the bacterial elicitor flagellin and represses basal immune responses, including flg22 (a conserved 22-amino acid peptide from flagellin)-induced MPK6 activation and defense-related gene expression [52] (Table 2). The inhibitory effect of MKKK7 on the attenuation of MPK6 activation might not be achieved through the direct modification of MAPKKs upstream of MPK6, but may occur via affecting the FLS2 complex by flg22-induced MKKK7 phosphorylation [52]. This scheme allows strict control of defense outputs to prevent erroneous or excessive immune activation.

In rice plants, the OsMKKK70-OsMKK4-OsMPK6 cascade regulates grain size and leaf angle [60]. In this signaling cascade, OsMKKK70 (a MEKK-like MAPKKK) shows in vitro kinase activity and physically interacts with its downstream kinase, OsMKK4, in yeast and tobacco cells, but does not phosphorylate OsMKK4 (Table 2). Intriguingly, OsMKKK70 promotes OsMPK6 phosphorylation in OsMKK4-dependent and -independent manners. As OsMKKK70 does not interact with OsMPK6, it is unlikely that OsMKKK70 functions as a scaffold protein tethering both OsMKK4 and OsMPK6 to promote OsMPK6 phosphorylation by OsMKK4. Thus, whether the OsMKKK70 kinase activity is involved in the regulation of the OsMKK4-OsMPK6 cascade and how OsMKK4-OsMPK6 is activated have to be further studied [60].

Altogether, these results illustrate the diversity of MEKK-like MAPKKKs with unconventional roles in the regulation of MAPK cascades and expand our understanding of the mechanisms underlying these MEKK-like MAPKKKs.

### 3.2. Special Roles of Plant Raf-like Kinases in the Regulation of MAPK Cascades

Plant Raf-like kinases have been grouped as MAPKKKs based on sequence similarity in some reports [1,3,6,7], while in others, they have been included in the tyrosine kinase-like (TKL) group and do not form a monophyletic group with metazoan Raf-like MAPKKKs [63]. Regardless of their classification, several studies have shown that plant Raf-like kinases do not function as genuine MAPKKKs that directly phosphorylate and activate downstream MAPKKs. For example, Arabidopsis CTR1, previously identified as a member of the Raf-like MAPKKKs, is involved in the regulation of ethylene signaling [64]. Yoo and coworkers found that constitutively active CTR1 repressed MPK3/MPK6 activity and located CTR1 upstream of the MKK9-MPK3/MPK6 cascade in ethylene signaling according to biochemical and genetic data [53] (Table 2). Subsequently, other reports have suggested that the MKK9-MPK3/MPK6 cascade regulates ethylene biosynthesis, and thus, might not function downstream of CTR1 [65,66,67,68,69,70]. More recently, the MKK1/MKK3-MPK3/MPK6 cascade, rather than the MKK9-MPK3/MPK6 cascade, was found to operate downstream of CTR1 in guard cell ethylene signaling (Table 2); and enhanced MPK3/MPK6 activation in the *ctr1* mutant was repeated [54], suggesting a negative role of CTR1 in the regulation of the MAPK cascade. CTR1 possesses kinase activity and phosphorylates MAPKK in vitro [71], but it might not affect MAPKK function in ethylene signaling through direct phosphorylation because EIN2 is the phosphorylation substrate of CTR1 in ethylene signaling [72,73]. Thus, CTR1 may indirectly regulate downstream MAPKKs.

Another Arabidopsis Raf-like MAPKKK, EDR1, negatively regulates innate immunity [74,75]. Furthermore, EDR1 is involved in the regulation of the MAPK cascade in an unusual manner in plant defense responses. EDR1 physically interacts with MKK4/MKK5 via its N-terminal domain and negatively regulates the accumulation of MKK4/MKK5-MPK3/MPK6 [55] (Table 2). Genetic data have shown that *edr1*-mediated Arabidopsis resistance to powdery mildew requires the MKK4/MKK5-MPK3/MPK6 cascade [55]. Therefore, EDR1 acts upstream of the MKK4/MKK5-MPK3/MPK6 cascade. Further results show that the accumulation of MKK4/MKK5 is associated with the phosphorylation of KEG, which encodes a RING E3 ubiquitin ligase and functions downstream of EDR1 in plant innate immunity [56,76]. In detail, KEG interacts with and ubiquitinates MKK4/MKK5, which in turn contributes to the degradation of MKK4/MKK5 by 26S proteasome; when EDR1 loses its function, KEG is phosphorylated and subsequently self-ubiquitinated and degraded, thus leading to the accumulation of MKK4/MKK5 [56]. These results suggest an uncanonical regulation of MAPKKs by a MAPKKK, wherein EDR1 affects the protein level of its downstream MKK4/MKK5-MPK3/MPK6 cascade through the modification of another protein that also functions downstream of EDR1. Given that *edr1*-mediated disease resistance requires LORELEI-LIKE GPI-ANCHORED PROTEIN 1 (LLG1), which is associated with the PRR complex and functions as a co-receptor of RLKs in the *mekk1*-*mkk1*/*mkk2*-*mpk4* cell death pathway [77,78], an alternative hypothesis is that EDR1 affects the downstream MKK4/MKK5-MPK3/MPK6 cascade via modification of the PRR complex, as MKKK7 does, thereby inhibiting PAMP-induced MAPK activation through modification of the PRR complex [52].

In addition to EDR1, another Arabidopsis Raf-like MAPKKK, MKD1, also contributes to plant immunity via its association with the MKK1/MKK5-MPK3/MPK6 cascade [57]. MKD1 interacts with MKK1/MKK5 and phosphorylates them in vitro (Table 2). The phosphorylation sites contained not only the canonical residues in the S/T-XXXXX-S/T motif, but also other amino acids beyond these sites. Loss of function of *MKD1* results in susceptibility to pathogens and a decreased degree of activation of MPK3/MPK6 in response to phytotoxins. As the *mkk1* mutant and *MKK5*RNAi transgenic plants mimic the *mkd1* mutants in response to pathogens, it was concluded that MKD1, MKK1/MKK5, and MPK3/MPK6 form a signaling cascade in plant responses against pathogens [57].

In addition, Arabidopsis Raf36 (a Raf-like MAPKKK), which possesses kinase activity and belongs to the Raf-like kinase group [79], was recently found to negatively regulate plant disease resistance by targeting MKK2 (a MAPKK) [58]. Raf36 interacts with MKK2 in plants, and phosphorylates MKK2 in vitro [58,59] (Table 2). The genetic data indicating that MKK2 positively regulates plant disease resistance and that MKK2 knockout compromises *raf36*-mediated disease resistance supports the Raf36-MKK2 signaling cascade. Indeed, Raf36 kinase activity is involved in the interaction with MKK2 and the modulation of plant disease resistance, although whether and how Raf36 kinase activity affects MKK2 function in the defense response remain unknown.

In rice plants, OsEDR1, the ortholog of Arabidopsis EDR1, is involved in the regulation of the MAPK cascade in rice disease resistance. OsEDR1 is a Raf-like MAPKKK and negatively regulates rice resistance to bacterial pathogens [80,81]. OsEDR1 physically interacts with but does not phosphorylate OsMPKK10.2, a MAPKK that positively regulates rice disease resistance and drought tolerance through the activation of different MAPKs [61,82] (Table 2). In *OsEDR1*-knock out mutants, the phosphorylation and kinase activity of OsMPKK10.2 toward its downstream MAPK OsMPK6, is enhanced [61]. Genetic data indicating that knocking-out *OsMPKK10.2* or *OsMPK6* compromised *osedr1*-mediated disease resistance place OsEDR1 upstream of the OsMPKK10.2-OsMPK6 cascade [61]. Remarkably, the enhanced activation of the OsMPKK10.2-OsMPK6 cascade by unidentified protein kinases may promote OsEDR1 degradation through direct phosphorylation of OsEDR1 by OsMPK6 [61]. Finally, OsEDR1 is considered a scaffold protein rather than a protein kinase in the regulation of the OsMPKK10.2-OsMPK6 cascade in rice disease resistance. Interestingly, it is noteworthy that *OsEDR1*, *OsMPKK10.2*, and *OsMPK6* are also involved in rice drought resistance [82,83], and whether they form a signaling cascade in drought resistance warrants further exploration.

Another Raf-like MAPKKK in rice plants, OsILA1, is also involved in the regulation of the MAPK cascade in rice resistance against bacterial pathogens. OsILA1 was initially identified as a regulator of the rice lamina joint through the interaction with and phosphorylation of CCCH-tandem zinc-finger transcription factors [84,85]. However, it was recently reported that OsILA1 negatively regulates rice resistance to bacterial pathogens via its association with the OsMPKK4-OsMPK6 cascade [62]. OsILA1 does not interact with OsMPKK4 in yeast cells but phosphorylates OsMPKK4 in vitro, mainly at the T34 site located in the N-terminal region (Table 2). Mutation of T34 to prevent its phosphorylation strongly inhibits OsMPKK4 phosphorylation by OsILA1 in rice protoplasts and increases the accumulation of OsMPKK4. Therefore, the phosphorylation of T34 might have affected the stability of OsMPKK4. However, it is difficult to conclude that OsILA1 phosphorylates and, in turn, destabilizes OsMPKK4. Acute observations are required to determine whether and why OsILA1 phosphorylates or destabilizes OsMPKK4 in rice cells without extracellular stimulus, as OsMPKK4 is required for rice plant development, including grain size formation [60,86,87,88]. Presumably, OsMPKK4 may coordinate rice plant development and responses to environmental stimuli via phosphorylation by different protein kinases.

Altogether, these results suggest that, although some Raf-like kinases possess kinase activities and can phosphorylate MAPKKs in vitro or even in planta, they do not function as canonical MAPKKKs to activate MAPK cascades. However, they do function as regulators of MAPK cascades. In fact, it was previously thought that classifying these kinases as Raf-like kinases might lead to the misconception that these kinases function as MAPKKKs [89]. Therefore, once a Raf-like kinase is determined to be associated with the MAPKK-MAPK cascade during a cellular response and can phosphorylate MAPKK in vitro or in planta, one must be cautious in establishing a link between Raf-mediated MAPKK phosphorylation and its function. Indeed, it is not surprising that a Raf-like MAPKKK phosphorylates MAPKKs in vitro or in planta at sites in the canonical S/T-XXXXX-S/T motif or beyond, because Raf-like kinases possess kinase activity and will probably yield a phosphorylation event when interacting with a MAPKK. Whether the phosphorylation occurs in vivo and contributes to the cellular response is essential and requires careful verification. In addition, it is necessary to note that Raf-like kinases regulate plant abiotic responses through direct phosphorylation of SNF1-related protein kinase2 instead of MAPKKs, thus providing firm phosphorylating substrates of Raf-like kinases in plant biological processes [68,90,91,92,93]. However, further studies are required to elucidate the role of Raf-like kinases in the regulation of MAPK cascades.

## 4. Activation of MAPKKs and MAPKs

A wealth of data, showing that MAPK cascades function in plant biological processes, support the hypothesis that MAPKK or MAPK is always phosphorylated and activated by the upstream MAPKKK or MAPKK, in a signaling cascade. However, several reports have indicated that plant MAPKKs or MAPKs can be phosphorylated or activated directly by protein kinases, rather than through MAPKKKs or MAPKKs, or by other alternative mechanisms which do not include MAPK components.

### 4.1. Direct Phosphorylation and Activation of MAPKKs by Non-MAPKKKs

In auxin-controlled cell division patterns during lateral root development, Arabidopsis transmembrane kinases TMK1 and TMK4, a group of RLKs, directly interact with and phosphorylate MKK4 and MKK5 [94] (Table 3). Biochemical and genetic data have revealed that auxin-induced MKK4/MKK5-MPK3/MPK6 phosphorylation is TMK1/TMK4-dependent, and that suppression of MKK4/MKK5 or MPK3/MPK6 expression leads to defects in lateral root development, as observed in *tmk1tmk4* double mutants [94]. Thus, the TMK1/TMK4-MKK4/MKK5-MPK3/MPK6 cascade signals in auxin-regulated cell division patterns presumably via sequential phosphorylation, although the phosphorylation sites of MKK4/MKK5 and the effect of TMK1/TMK4 phosphorylation of MKK4/MKK5 on its kinase activity are unclear.

In rice bacterial-pathogen resistance, OsMPKK10.2 can be phosphorylated and activated by non-MAPKKKs [61]. It has been shown that during pathogen infection, OsMPKK10.2 is phosphorylated at many sites that are not located in the S/T-XXXXX-S/T activation motif. Specifically, phosphorylation of the amino acid residue, S304, located at the C-terminal domain, enhances OsMPKK10.2 kinase activity toward its downstream MAPK OsMPK6, thereby promoting rice disease resistance (Table 3). Nonetheless, the specific interplay of phosphorylation between S304 and the S/T-XXXXX-S/T motif awaits elucidation. Additionally, the upstream kinase responsible for OsMPKK10.2 S304 phosphorylation has not been identified. It is important to unravel these issues for a thorough understanding of OsMPKK10.2 activation by different protein kinases.

In the abscisic acid (ABA) signaling-pathway in rice, another MAPKK, OsMKK1, is directly phosphorylated and activated by OsDMI3, a Ca^2+^/calmodulin-dependent protein kinase (CCaMK) [95] (Table 3). ABA-induced OsDMI3 phosphorylates the amino acid residue T25 located at the N-terminal domain of OsMKK1 but not the canonical S/T-XXXXX-S/T motif. Phosphorylation of T25 increases OsMKK1 kinase activity to its downstream MAPK, OsMPK6 (named OsMPK1 in the original article). Simultaneously, the two canonical sites in the S/T-XXXXX-S/T motif are also phosphorylated in response to ABA treatment. These two phosphorylation events do not affect each other. Therefore, it seems likely that the phosphorylation of OsMKK1 by MAPKKKs and OsDMI3 coordinates and facilitates the adaptation of rice plants to abiotic stress.

### 4.2. Activation of MAPKs

In the plant MAPK cascade, MAPK is typically activated through MAPKK-mediated dual phosphorylation at T and tyrosine (Y) residues within the TXY motif [7]. However, plant MAPKs can also be activated in alternative ways.

For instance, in Arabidopsis, NDPK2, an NDP kinase, is associated with H_2_O_2_-mediated MAPK signaling [96]. NDPK2 binds to MPK3 in vitro; it does not phosphorylate MPK3, but enhances MPK3 phosphorylation ability against myelin basic protein (MBP, always used as a common phosphorylation substrate of MAPKs) (Table 3), thus suggesting direct activation of MPK3 by NDPK2 through an unknown mechanism which does not require MAPKKs [96].

Furthermore, Arabidopsis MPK6 can also be activated by unusual modes. For example, MPK6 activation is induced when Arabidopsis is exposed to NaCl [106]. Further analysis showed that NaCl treatment increases the generation of phosphatidic acid (PA), which in turn binds to MPK6 and facilitates MPK6 phosphorylation ability toward its substrate [97] (Table 3). Furthermore, PA can also bind to MPK3 and MPK6 when Arabidopsis undergoes submergence; moreover, this binding promotes MPK3/MPK6 kinase activity towards their substrates to modulate plant tolerance to submergence [98] (Table 3). As the kinase activity of the upstream kinase MKK5 is also enhanced by PA, it is possible that PA activates a MAPK cascade consisting of MKK5 and MPK3/MPK6 [98]. Additionally, PA reportedly binds directly to CTR1 and inhibits its kinase activity to coordinate ethylene signaling and the submergence response [107,108]. The ortholog of AtMPK6 in rice plants, OsMPK6, is also involved in the regulation of rice response to salt stress. When rice plants are exposed to salt stress, a lectin receptor-like kinase SIT1 is induced, which in turn interacts with and phosphorylates OsMPK6, which finally results in the excess accumulation of reactive oxygen species and, thus, in salt sensitivity [104] (Table 3). Although OsMPK6 phosphorylation sites by SIT1 are unknown, phosphorylation of the TXY motif, which is typically conferred by MAPKKs, is positively associated with SIT1 kinase activity, suggesting a direct link between OsMPK6 activation and SIT1 kinase activity in rice response to salt stress.

In Arabidopsis responses to cold stress, MPK4 is required in alleviating cell damages [99]. Later, it was found that hydrogen sulfide (H_2_S) modifies cysteines of MPK4 by persulfidation, and promotes MPK4 kinase activity to enhance Arabidopsis resistance to cold stress [100]. Persulfidation of a protein kinase can alter its structure and improve transfer efficiency of phosphate form ATP to target phosphorylation sites, thus enhancing the phosphorylation level [109]. However, how persulfidation promotes MPK4 activity remains unknown and requires further elucidation.

Other MAPKs have also been found to be directly activated in plants without the intervention of MAPKKs. For example, Arabidopsis MPK9, which is also induced by salt stress, autophosphorylates its TXY motif and other sites in the C-terminal domain in an MPKK-independent manner [101] (Table 3), thereby giving rise to the intriguing question: How do environmental signals flow to MPK9? It is not clear whether the aforementioned MAPK-activation modes operating under salt stress operate in the MPK9-mediated salt stress response as well.

Several calmodulins in Arabidopsis act as Ca^2+^-binding proteins (CaMs) bound to MPK8 in a Ca^2+^-dependent manner [102]. This binding does not modify the phosphorylation of the TXY motif in MPK8 by upstream MAPKKs, but enhances MPK8 kinase activity toward MBP (Table 3). The two modes of MPK8 activation by CaMs and MAPKKs are independent, but might be reciprocal in plant wounding responses [102].

Similarly, Ca^2+^ has been found to be associated with OsMPK3 (also known as OsMPK5) in rice plants [105]. During rice blast resistance, the Ca^2+^-dependent protein kinase CPK18 interacts with and phosphorylates OsMPK3 (Table 3). Such phosphorylation takes place at the T14 and T32 sites located in the N-terminus, resulting in OsMPK3 activation, which then represses defense-related gene expression and leads to the inhibition of rice blast resistance [105]. Seemingly, the phosphorylation and activation of OsMPK3 by CPK18 does not affect TEY motif phosphorylation, and thus, is MAPKK-independent. Additionally, in ABA signaling in maize, silencing of *ZmCPK11* decreased ZmMPK5 (the orthologue of rice OsMPK3) expression and kinase activity [110], leading to the indication of conserved mechanisms of MAPK activation by CPKs in plants.

Recently, MPK15 was found to confer Arabidopsis resistance to fungal pathogens [103]. MPK15 is phosphorylated in response to pathogen infection and PAMP stimulation. Phosphorylation by unknown kinases at S511, located in the C-terminal tail, is RLCK-dependent and activates MPK15, which then contributes to plant resistance (Table 3). MPK15 is also self-phosphorylated at S511 (Table 3). Complete activation of MPK15 requires both S511 phosphorylation and TXY motif phosphorylation, suggesting a possible mutual potentiation scheme between the two phosphorylation modes.

## 5. Conclusions and Perspectives

**Figure 1:**
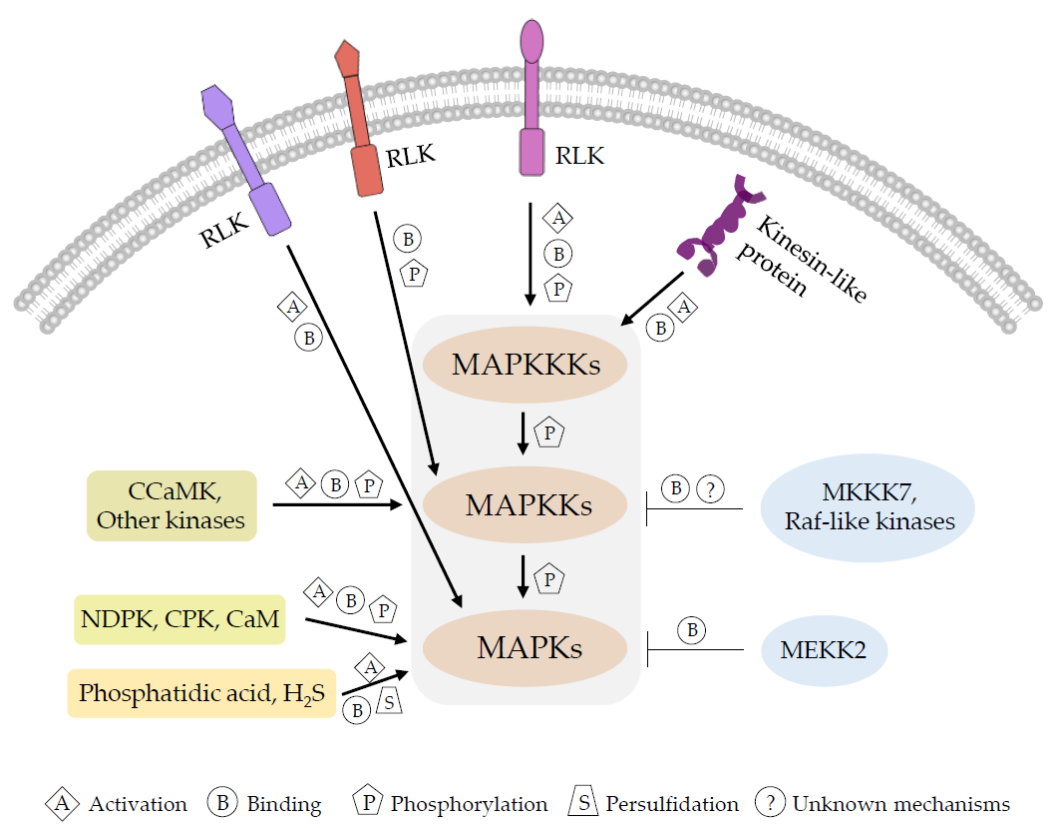
Activation modes of plant MAPK cascades. Each tier of the signaling cascade can be activated by different modes. MAPKKKs can be activated by kinesin-like proteins and receptor-like kinases (RLKs) through direct binding and/or phosphorylation. MAPKKs can also be directly activated by RLKs, or other protein kinases such as calcium/calmodulin-dependent protein kinase (CCaMK), through binding or phosphorylation. The activation modes of MAPKs vary considerably. Proteins including NDP kinase (NDPK), calcium-dependent protein kinase (CPK), and calcium-binding protein (CaM) activate MAPKs through binding and/or phosphorylation. Phosphatidic acid and H_2_S promote MAPKs activation through direct binding and persulfidation, respectively. It is necessary to note that some MAPKKKs, especially some Raf-like kinases, do not function as genuine MAPKKKs, although they are still involved in the regulation of MAPK cascades. Arrows and T lines indicate positive and negative regulations, respectively.

Mounting evidence proves that the number of MAPK members have expanded in land plants with functions in complex growth regulation and adaptation to dynamic environmental conditions [1,111,112]. A crucial question is how MAPK cascades are activated or regulated when plants face different stimuli. Although the detailed mechanisms remain unclear, the current data listed above suggest that the activation modes of each member of the three-tiered system vary (Figure 1).

As many MAPK cascades are genetically located downstream of RLKs [9,10,113], the activation of MAPKKKs appear to associate tightly with RLKs in most cases. Some MAPKKKs in the MAPK cascade can be directly phosphorylated and activated by RLKs to activate downstream MAPK members. However, in most cases, the activation of MAPKKKs has not been entirely clarified, especially at the biochemical level. Moreover, the principles or rules of MAPKKK phosphorylation and activation are largely unknown and require further elucidation.

For other MAPK members referring to MAPKKs and MAPKs, various proteins and metabolites are involved in their regulation. Many MAPKKKs or non-MAPKKKs affect MAPKKs protein accumulation or kinase activity directly or indirectly, and many non-MAPKKs or metabolites activate MAPKs through direct phosphorylation or binding, showing unusual modes of action that are independent of or coordinated with sequential phosphorylation mechanisms. These non-traditional regulation modes of MAPK cascades allow for the coordinated control of plant growth regulation or stress responses and require further in-depth analysis.

## Figures and Tables

**Table 1:** Representative activation of MAPKKKs by various proteins in plants.

Plant Species	Proteins	Protein Types	Target MAPKKKs	Regulatory Mechanisms	Biological Functions	References
*Nicotiana tabacum*	NACK1/2	kinesin-like proteins	NPK1	Physical interaction and activation	Plant cytokinesis	[11,12,13]
*Arabidopsis thaliana*	SSP	RLCK	YDA	Physical interaction and activation	Plant embryogenesis	[20,21,22]
	CRLK1	RLK	MEKK1	Physical interaction, direct phosphorylation	Plant cold response	[23,24]
	RLCK VII-4 members, BSK1	RLCK	MAPKKK3/5, MEKK1	Direct phosphorylation and activation	Plant immunity	[25,26,27]
*Oryza sativa*	OsRLCK185	RLCK	OsMAPKKK18/24	Direct phosphorylation and activation	Plant immunity	[28,29]
*Solanum lycopersicum*	SlMai1	RLCK	MAPKKKα	Physical interaction and activation	Plant immunity	[30]

**Table 2:** Uncanonical regulation of MAPK members by MAPKKKs in plants.

	MAPKKKs	Subgroup	Target MAPK Members	Regulatory Mechanisms	Biological Functions	References
*Arabidopsis thaliana*	MEKK2	MEKK	MPK4	Physical interaction and direct inhibition	Plant immunity	[30,46,47,48,49,50,51]
	MKKK7	MEKK	MPK6	Indirect inhibition through PRR complex	Plant immunity	[52]
	CTR1	Raf	MKK9-MPK3/MPK6, MKK1/MKK3-MPK3/MPK6	Indirect inhibition	Plant ethylene signaling	[53,54]
	EDR1	Raf	MKK4/MKK5	Physical interaction, inhibition	Plant immunity	[55,56]
	MKD1	Raf	MKK1/MKK5	Physical interaction, in vitro phosphorylation	Plant immunity	[57]
	Raf36	Raf	MKK2	Physical interaction, in vitro phosphorylation	Plant immunity	[58,59]
*Oryza sativa*	OsMKKK70	MEKK	OsMKK4	Physical interaction and activation	Grain size and leaf angle	[60]
	OsEDR1	Raf	OsMPKK10.2	Physical interaction and inhibition	Plant immunity	[61]
	OsILA1	Raf	OsMPKK4	In vitro phosphorylation, inhibition	Plant immunity	[62]

**Table 3:** Uncanonical activation of MAPK members in plants.

	Factors	Category	Target MAPK Members	Regulatory Mechanisms	Biological Functions	References
*Arabidopsis thaliana*	TMK1/4	RLK	MKK4/5	Physical interaction, phosphorylation	Auxin-regulated cell division	[94]
	NDPK2	NDP kinase	MPK3	Activation through binding	Cellular redox	[96]
	Phosphatidic acid	Metabolite	MPK3/6	Activation through binding	Salt stress and submergence responses	[97,98]
	H_2_S	Gas	MPK4	Activation through persulfidation	Cold stress response	[99,100]
	MPK9	MAPK	MPK9	Autophosphorylation	Salt stress responses	[101]
	CaM	Ca^2+^-binding protein	MPK8	Activation through binding	Plant wounding response	[102]
	Unknown	Protein kinase	MPK15	Autophosphorylation, trans-phosphorylation	Plant immunity	[103]
*Oryza sativa*	Unknown	Protein kinase	OsMPKK10.2	Direct phosphorylation and activation	Plant immunity	[61]
	OsDMI3	CCaMK	OsMKK1	Direct phosphorylation and activation	ABA signaling	[95]
	SIT1	Lectin RLK	OsMPK6	Physical interaction, in vitro phosphorylation, activation	Salt stress responses	[104]
	CPK18	Ca^2+^-dependent protein kinase	OsMPK3	Direct interaction, phosphorylation and activation	Plant immunity	[105]

## Data Availability

Not applicable.
